# RENAL INFECTION VS. INFARCTION: A CHALLENGING DIAGNOSIS AND A RARE CASE REPORT

**DOI:** 10.51894/001c.122886

**Published:** 2024-08-30

**Authors:** Spencer Gilbert, Bashar Hammad, Heba Al Sabbagh, M. Ammar Hatahet

**Affiliations:** 1 College of Osteopathic Medicine Michigan State University

## 32

### INTRODUCTION

Flank pain is a common reason patients seek medical attention. Two specific etiologies are pyelonephritis and renal infarction. Pyelonephritis is a bacterial infection of the renal pelvis and parenchyma that typically presents with flank pain, urinary symptoms and systemic signs. Renal infarction is ischemic damage related to decreased renal perfusion, often due to thromboembolism or in-situ thrombosis, with 1/3 of cases being idiopathic.

### CASE DESCRIPTION

Here we report a case of a 37-year-old female who presented to the emergency department with chief complaint of sudden onset right flank pain that was sharp, constant, and radiating to her right lower quadrant. Physical exam showed a positive Lloyd’s sign and right upper quadrant tenderness. Labs were pertinent for leukocytosis, elevated LDH level, normal creatinine and a negative UA. CT abdomen with contrast showed multiple wedge-shaped hypodensities on the right, and the patient was started on antibiotics for suspected pyelonephritis. Subsequent CTA showed atherosclerosis of the abdominal aorta and remonstrated the wedge-shaped hypodensities of the right kidney without abnormalities of the renal arteries, raising concern for superimposed renal infarction. The patient was concurrently started on anticoagulation. Echo showed no abnormalities. On day 3 of admission, the patient showed clinical improvement and was discharged with outpatient follow-up.

### DISCUSSION

Due to the patient’s presenting symptoms, lab and imaging findings, the question was raised regarding a differential between pyelonephritis and renal infarcts as both can present with wedge shaped hypodensities on CT. In about 50% of cases of renal infarcts, the cortical rim sign can be observed, with enhancement of the rim of the renal cortex. In this case, CT demonstrated wedge-shaped hypodensities without a positive cortical rim sign, causing ambiguity. This case illustrates the benefits and limitations of imaging for both renal infarcts and pyelonephritis in that although the findings on the initial CT aided in rapidly narrowing the differential diagnosis, it was limited in its sensitivity to distinguish between the two disease states, highlighting the importance of a holistic approach when formulating a differential diagnosis and the potential implication of using anticoagulation and antibiotics concurrently when renal infarction vs infection cannot be clearly delineated.

